# EWS/FLI1 Target Genes and Therapeutic Opportunities in Ewing Sarcoma

**DOI:** 10.3389/fonc.2015.00162

**Published:** 2015-07-20

**Authors:** Florencia Cidre-Aranaz, Javier Alonso

**Affiliations:** ^1^Unidad de Tumores Sólidos Infantiles, Área de Genética Humana, Instituto de Investigación de Enfermedades Raras, Instituto de Salud Carlos III, Madrid, Spain

**Keywords:** Ewing sarcoma, EWS/FLI1, DAX-1, GLI1, FOXO1, FOXM1, CCK, LOX

## Abstract

Ewing sarcoma is an aggressive bone malignancy that affect children and young adults. Ewing sarcoma is the second most common primary bone malignancy in pediatric patients. Although significant progress has been made in the treatment of Ewing sarcoma since it was first described in the 1920s, in the last decade survival rates have remained unacceptably invariable, thus pointing to the need for new approaches centered in the molecular basis of the disease. Ewing sarcoma driving mutation, *EWS–FLI1*, which results from a chromosomal translocation, encodes an aberrant transcription factor. Since its first characterization in 1990s, many molecular targets have been described to be regulated by this chimeric transcription factor. Their contribution to orchestrate Ewing sarcoma phenotype has been reported over the last decades. In this work, we will focus on the description of a selection of EWS/FLI1 targets, their functional role, and their potential clinical relevance. We will also discuss their role in other types of cancer as well as the need for further studies to be performed in order to achieve a broader understanding of their particular contribution to Ewing sarcoma development.

## Introduction

Ewing sarcoma is a rare tumor that arises mainly in the bones of children and adolescents. Despite the improvements in treatment achieved during the last decades, survival rates have remained unacceptably low, even in patients with localized disease, since a great proportion of Ewing sarcoma tumors are refractory to conventional treatment and relapses are frequent (1). In addition, approximately 25% of cases present disseminated disease at diagnosis, which have a very poor prognosis (2). Thus, there is an urgent need for new targeted therapies that may offer a higher efficiency and less adverse effects than the conventional chemo/radiotherapies that are used nowadays.

In this sense, understanding the molecular basis of Ewing sarcoma pathogenesis provides key information that may help to design new targeted biological therapies. Ewing sarcomas are characterized by chromosomal translocations that fuse the *EWSR1* gene to some members of the ETS family of transcription factors (3), being FLI1 the most frequently implicated [t(11;22)(q24;q12)] (4). The EWS/FLI1 fusion protein is an aberrant transcription factor that is essential for Ewing tumor development, since it regulates the expression of multiple target genes and governs the oncogenic processes that lead to malignant transformation of a yet undefined cancer precursor cell. Provided that the oncogenic properties of EWS/FLI1 rely on its capability to induce or repress specific target genes, these target genes can likewise offer interesting opportunities to identify new targeted therapies.

In the past years, an important effort to identify EWS/FLI1 genes functionally relevant for Ewing sarcoma pathogenesis has been carried out. As a consequence, many genes that play an important role in Ewing sarcoma have been identified (5–17). This has revealed some key molecular pathways involved in Ewing pathogenesis, and more importantly it has provided new molecular targets.

A comprehensive discussion of all EWS/FLI1 target genes identified to date and their implications in targeted therapy is beyond the scope of this review. Thus, here we have focused on a selection of six EWS/FLI1 target genes that, in our opinion, can represent attractive opportunities for future studies that may lead to discovering new therapeutic approaches. This selection takes into account the presence of significant data – in Ewing or in other systems – regarding potential therapeutic applications. Four genes encode for transcriptional regulators while the other two encode for secreted proteins.

## Transcriptional Regulators

### DAX-1 (NR0B1)

*DAX-1* is a gene that belongs to the super family of nuclear receptors (official name *NR0B1*, standing for Nuclear Receptor Subfamily 0, Group B, Member 1). Nuclear receptors are transcription factors that undergo activation upon binding of small ligands such as retinoic acid or steroids. However, there is no known ligand for DAX-1, and thus we refer to it as an orphan nuclear receptor. Germline mutations in this gene are the cause of dosage-sensitive sex reversal (DSS) in XY individuals and adrenal hypoplasia congenital (AHC), which is characterized by adrenal insufficiency, and hypogonadotropic hypogonadism in males. DAX-1 is a master regulator of steroidogenesis that negatively regulates the steroidogenic factor 1 (SF1), an important transcriptional activator of genes involved in steroid hormone production (18, 19). In addition, DAX-1 plays an important role in several biological processes such as osteoblast differentiation (20), ion homeostasis and transport, lipid transport, or skeletal development (21) among others. More recently, DAX-1 has been involved in the maintenance of mouse embryonic stem cell pluripotency through regulation of stem cell genes like *Oct-3/4* (22–24).

Given that DAX-1 function is mainly linked to steroidogenesis, it was surprising to find this gene associated to Ewing sarcoma, a tumor with no known relationship with steroidogenic tissues. Gene expression profiles performed in two heterologous cell models ectopically expressing EWS/FLI1 (HEK293 and HeLa cells) demonstrated that DAX-1 was specifically induced by EWS/FLI1, but not by wildtype FLI1 (25). In addition, it was shown that DAX-1 was highly expressed in Ewing sarcoma cell lines and tumors, while it was not expressed in other pediatric tumors such as rhabdomyosarcoma or neuroblastoma. Finally, DAX-1 expression was demonstrated to depend on EWS/FLI1 expression in the A673 Ewing sarcoma cell line upon EWS/FLI1 knockdown. An independent study showed similar findings, confirming that DAX-1 is a target of the EWS/FLI1 oncoprotein (26).

Several functional studies have demonstrated that DAX-1 plays a critical role in Ewing sarcoma pathogenesis: DAX-1 knockdown impairs Ewing sarcoma cell proliferation, G1 cell arrest induction, inhibits anchorage independent growth of colonies in soft agar, and drastically inhibits growth of xenotransplanted tumor cells in immunodeficient mice (9, 25, 26). These results are highly consistent since they were obtained in independent laboratories, using several Ewing sarcoma cell lines (TC71, EWS502, and A673) and different gene knockdown technologies (i.e., transient retrovirus infection or inducible expression of EWS/FLI1 shRNAs). Interestingly, characterization of the gene expression profile regulated by DAX-1 in Ewing sarcoma cell lines has also provided interesting findings regarding the function of DAX-1 in Ewing sarcoma. These studies showed that a significant percentage of the genes regulated by EWS/FLI1 in Ewing sarcoma cells are also under the control of DAX-1, reinforcing the importance of DAX-1 in Ewing sarcoma pathogenesis. In fact, two independent works demonstrated that EWS/FLI1 and DAX-1 transcriptional profiles share a significant number of genes, suggesting that DAX-1 not only contributes to the EWS/FLI1 transcriptional signature but also that there is a hierarchy controlled by EWS/FLI1 and in which some genes, such as *DAX-1*, can play a more prominent role (9, 27). The study of the mechanism through which EWS/FLI1 upregulates DAX-1 expression in Ewing sarcoma cells revealed an unexpected finding: EWS/FLI1 directly interacts with *DAX-1* promoter through binding to a GGAA-rich sequence (9, 28). This motif resulted to be a polymorphic microsatellite located in the *DAX-1* promoter. It has been demonstrated that EWS/FLI1 binds similar sequences located in the promoters of other EWS/FLI1 target genes, indicating that this mechanism of gene transcriptional activation is frequently used by EWS/FLI1 to regulate the expression of some oncogenic genes (28) [i.e., *Caveolin-1* (*CAV1*) (7), *glutathione S-transferase M4* (*GSTM4*) (29), *FCGRT* (*Fc fragment of IgG, receptor, transporter, alpha*), *FVT1/KDSR* (*3-ketodihydrosphingosine reductase*)or *ABHD6* (*Abhydrolase Domain-Containing Protein*) (30)]. The fact that *DAX-1* expression is regulated through a polymorphic repeat of the GGAA motif raised the question if the number of repeats could be somehow linked to the level of *DAX-1* expression and, as a consequence, to the malignant phenotype of Ewing sarcoma. Several biochemical studies demonstrated a relationship between the number of GGAA repeats and the degree of promoter activation, indicating that it was necessary a minimum of nine repeats to obtain a response to EWS/FLI1 (30). However, the attempts to establish a relationship between the length of the microsatellite located in *DAX-1* promoter and the clinical prognosis have raised contradictory results. For instance, GGAA microsatellites were longer in African populations, which are known to have a lower incidence of Ewing sarcoma but a worse overall survival when compared to European populations (31, 32). Conversely, in another study based on 112 patients, the length of the DAX-1 microsatellite showed no influence on clinical outcomes (33).

Taking into account all these results, *DAX-1* can be considered as one of the most relevant EWS/FLI1 gene targets. The fact that DAX-1 expression results essential for EWS/FLI1-mediated oncogenesis opens the possibility, at least in theory, to consider *DAX-1* targeting as an attractive therapeutic approach in Ewing sarcoma. As a consequence, a more profound understanding of the functions that DAX-1 exerts in Ewing sarcoma and the molecular mechanism involved in them can provide new clues on how to interfere with its expression or function in this cancer (34).

DAX-1 is located in the nucleus of Ewing sarcoma cells, where it presumably interacts with other transcription factors and cofactors to regulate downstream target genes that are important for oncogenesis. Interestingly, a combination of biochemical and gene expression profile experiments leads to the observation that EWS/FLI1 and DAX-1 interact physically. Specifically, it was found that both the amino- and carboxyl-termini of DAX-1 interacted with EWS/FLI1 (27). This result opens the attractive possibility that interfering EWS/FLI1-DAX-1 interaction could lead to new therapeutic opportunities. To go forward in this line of work, it would be necessary to finely map the regions involved in this interaction in order to design small molecules with the ability to block it. Since DAX-1 and EWS/FLI1 interaction could be necessary for full EWS/FLI1-mediated oncogenesis, disturbing it could be therapeutically valuable.

DAX-1 has been shown to interact in different cellular contexts with a variety of transcriptional regulators, mainly corepressors. For example, DAX-1 interacts with Alien corepressor through its silencing domain and this interaction has been shown to be important for AHC pathogenesis (35). DAX-1 has also been shown to interact directly with the androgen receptor, NR3C4, inhibiting its activation (36) and other partners such as NR5A1 (37) and ESRRγ (38). To date, a systematic analysis of the protein–protein interactions in which DAX-1 is involved in Ewing sarcoma cells and the role that these interactions can play in Ewing sarcoma pathogenesis has not been carried out. Experiments focused on identifying and characterizing these interactions could provide clues for designing synthetic drugs to target them. On the other hand, it has been shown that DAX-1 C-terminal domain contains a potent transcriptional repressor domain that, when altered by mutations in AHC patients, impairs its nuclear localization, and therefore its transcriptional activity (39), suggesting that there is a potential field for developing drugs to modulate DAX-1 subcellular localization and consequently its function.

**Figure 1:**
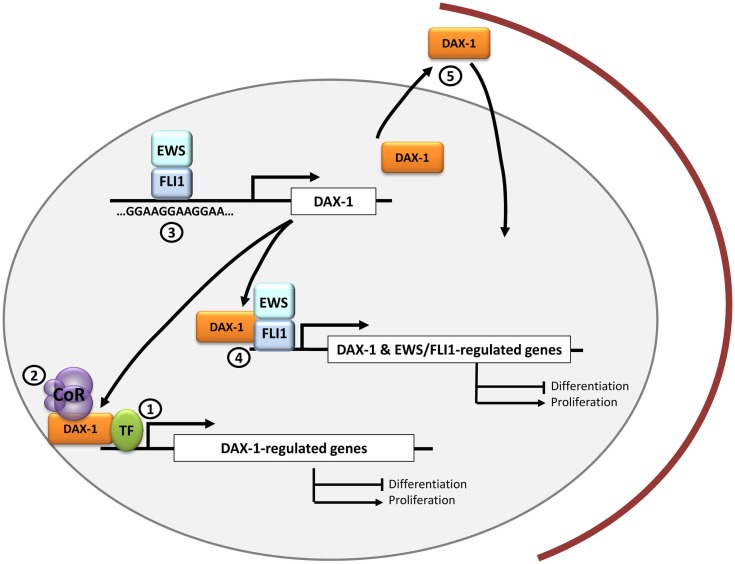
**DAX-1 and therapeutic opportunities in Ewing sarcoma**. DAX-1 expression is upregulated by EWS/FLI1 in Ewing sarcoma cells through a direct interaction with a polymorphic GGAA microsatellite located in the *DAX-1* promoter. Since DAX-1 expression is essential for EWS/FLI1-mediated oncogenesis, it is still necessary to ascertain if it interacts with other transcription factors (1) and/or co-repressors (2) in the nucleus of Ewing sarcoma cells. This could open new therapeutic approaches for designing molecules to target these interactions. Potential therapeutic targets may be molecules that prevent EWS/FLI1 binding to the GGAA-rich motifs in *DAX-1* promoter (3) or drugs directed toward the EWS/FLI1-DAX-1 interaction, whose concurrence could be necessary to regulate the expression of certain genes (4). Finally, DAX-1 C-terminal domain can impair DAX-1 nuclear localization when altered so it could be potentially targeted to modulate its subcellular localization and thus its function (5).

As with any new drug, the possible side effects of a new therapeutic approach must be also taken into consideration. For instance, prolonged DAX-1 blockage may lead to disequilibrium in steroid hormones production, which could lead to Cushing-like syndrome (40). These hypothetical complications, compared with the severity of Ewing sarcoma itself, would be perfectly assumable. One theoretical advantage of using therapeutic approaches targeting *DAX-1* is that this gene is only expressed in a limited number of tissues, mainly in adrenal gland and testis, and probably DAX-1 targeting will only affect these organs. In summary, although there are currently no drugs able to target DAX-1 and block its function, studies intended to understand its structure, its mechanism of interaction with other transcriptional (co)factors, and the identification of other protein–protein interactions in the Ewing sarcoma context could provide new insights to design new therapeutic molecules (Figure 1).

### GLI1

GLI1 (*Glioma-Associated Oncogene Homolog 1)* is a transcription factor belonging to the Kruper family of zinc finger proteins. GLI1 is a component of the canonical Hedgehog pathway: extracellular Sonic Hedgehog (Shh) binds to the PTCH receptor causing the liberation of Smooth (SMO) from the PTCH-SMO complex. Subsequently, activated SMO releases GLI1 from the complex that it forms with Suppressor of Fused (SUFU), which permits GLI1 nuclear translocation where it regulates gene transcription of genes involved in normal cell growth and differentiation such as the embryonic pattern formation (41). Although this pathway is mainly active during embryogenesis, it remains active in some adult tissues, where it is involved in homeostasis and stem-cell maintenance (42, 43).

Zwerner et al. described an association between EWS/FLI1 and GLI1 in Ewing sarcoma cells. They showed that NIH3T3 cells expressing *EWS/FLI1* presented the expected malignant phenotype concomitantly with augmented levels of GLI1 (44). Moreover, when GLI1 expression was knocked-down by RNA interference, the transformed phenotype was reduced (demonstrated by a decrease in the anchorage independent growth) indicating that GLI1 plays an important role in the maintenance of the malignant phenotype induced by EWS/FLI1. Interestingly, SUFU overexpression, which is expected to inhibit GLI1, also produced similar effects in NIH3T3 cells. In TC32 Ewing sarcoma cells, EWS/FLI1 knocking down using RNA interference produced a reduction in *GLI1* expression levels. Also, ChIP studies demonstrated that GLI1 is a direct target of EWS/FLI1 (45). Moreover, when a shRNA against GLI1 was used in the Ewing sarcoma cell line TC32, the transformed phenotype was inhibited (measured by reduction in anchorage independent growth) (44). Interestingly, and in contrast with what it is usually observed in other types of cancer, GLI1 deregulation in Ewing sarcoma is independent of Shh since its activation did not produce phenotypic changes nor did a pharmacological blockage of SMO using cyclopamine (an inhibitor of Shh signaling by direct binding to SMO) (45).

Subsequently Joo et al. (46) showed that Ewing primary tumors expressed high levels of GLI1. These authors also confirmed using RNAi that the expression of GLI1 in Ewing sarcoma cells (TC71) is dependent on EWS/FLI1 and that GLI1 expression was relevant for the maintenance of the transformed phenotype. Strikingly, re-analysis of gene expression profiles showed that genes that were traditionally thought to be transcriptionally modulated by EWS/FLI1, such as *NKX2.2*, *Patched (PTCH)* or *GAS1*, were indeed dependent on GLI1 expression, meaning that the gene expression network regulated by EWS/FLI1 holds a hierarchy in which GLI1 has a prominent role.

Deregulation of the Shh–GLI1 pathway has been showed to lead to tumorigenesis and aggressive phenotypes (progression, metastasis and therapeutic resistance) of numerous cancer types such as basal cell carcinomas (47), colorectal carcinoma (48), breast cancer (49), and bone and soft tissue sarcomas (50).

Given the importance of Shh–GLI1 pathway in cancer, some therapeutic approaches, focused on the blocking of this pathway, have been developed over the years. One of these strategies consisted in searching for small molecule inhibitors of the pathway. Thus, Shh–GLI1 pathway inhibitors, such as cyclopamine, have been successfully tested in some cancer types such as medulloblastoma (51), pancreatic adenocarcinoma (52), small-cell lung cancer (SCLC) (53), gastric adenocarcinomas (54), and esophageal cancer (55).

The fact that GLI1 expression is constitutively induced by EWS/FLI1 in Ewing sarcoma suggests that drugs acting upstream GLI1 will be ineffective in blocking this pathway in this cancer. In agreement with this, cyclopamine treatment of Ewing sarcoma cells would have no effect on the malignant characteristics of Ewing sarcoma cells. For this reason, efforts should be directed toward developing and studying drugs targeting GLI1 expression or function directly. In this sense, arsenic trioxide (ATO), an old drug recently reintroduced in the repertoire of anticancer drugs, has been found to inhibit cell growth by targeting GLI proteins (56). In the specific case of Ewing sarcoma, ATO presented cytotoxicity in cell lines with upregulated GLI1 expression (TC-71, SKES and A4573), and curbed xenograft growth performed with TC-71 cells (57). ATO was also found to inhibit Ewing cells (RDES and A673) migration and invasiveness, thus implying that it could also have a therapeutic effect on metastasis (58). Of note, ATO has already been tested in combination with other chemotherapeutic drugs (etoposide and paclitaxel) in a preliminary study that included Ewing sarcoma and metastatic osteosarcoma patients where tumor growth was controlled in 75% of cases (59). However, since Ewing sarcoma is mainly a pediatric cancer, it is necessary to further investigate its effects and to be prudent when designing clinical studies given the roles of the Shh–GLI1 pathway in development.

Finally, it has been described a correlation between GLI1 expression levels and/or prognosis and recurrence in some cancer types. For instance, in a study comprising 25 clinical samples of colorectal carcinoma, Shh expression was found upregulated and, interestingly, when GLI1 expression was analyzed by *in situ* hybridization, it was mainly found in the malignant crypts of adenocarcinomas (48). Also, it has been described a positive correlation between GLI1 expression and tumor grade and/or lymph node status, pointing to a role of GLI1 in metastasis. Some examples are breast cancer, where high GLI1 expression measured in a TMA containing 204 tumor samples was associated with poor prognosis and progressive stages of disease (49) or bone and soft tissue sarcomas, where higher GLI1 expression correlated with more aggressive outcomes (50). In the specific case of Ewing sarcoma, these studies remain to be performed, especially considering that a deeper knowledge on this field could lead to a more efficient patient stratification that could help improve treatment.

**Figure 2:**
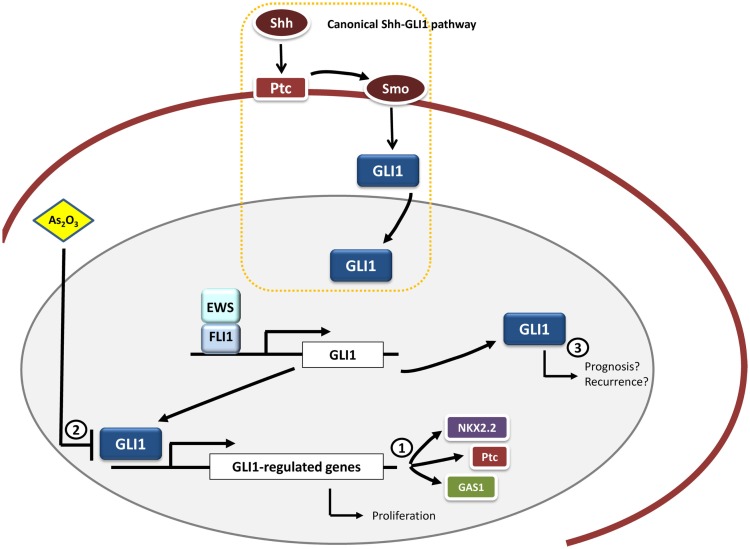
**GLI1 and therapeutic opportunities in Ewing sarcoma**. *GLI1* is an upregulated direct target gene of EWS/FLI1 in Ewing sarcoma cells. Functional studies have demonstrated that GLI1 expression is relevant for the maintenance of the transformed phenotype in this system. Moreover, some genes transcriptionally modulated by EWS/FLI1 depend on GLI1 expression, including *NKX2.2, PATCH*, and *GAS1* (1). Therapeutic opportunities may include the use of molecules capable of inhibiting GLI1-mediated transcription, such as arsenic trioxide (As_2_O_3_) (2). Also it may be interesting to ascertain the possible link between GLI1 expression pattern and prognosis in Ewing sarcoma given that this correlation between GLI1 expression and bad prognosis exists in other tumor types (breast cancer and bone and soft tissue sarcomas) (3).

There is still an urgent need for further functional studies that can ascertain the exact role of this pathway in Ewing sarcoma development and progression. These studies could help to synthesize new compounds or small molecules that could target GLI1 with better efficacies either alone or in combination with normal chemotherapeutic treatments (Figure 2).

### Forkhead box (FOX) of transcription factors

Forkhead box proteins are an extensive family of transcriptional regulators that share a common DNA binding domain (the forkhead domain). There are 19 subgroups (FOXA to FOXS) organized on the basis of sequence homology inside and outside the forkhead domain. FOX proteins regulate gene networks that are involved in cell cycle progression, proliferation, differentiation, metabolism, senescence, survival, or apoptosis (60). Thus, it is not strange that these transcription factors have been shown to have roles in cancer. Interestingly, some members of this family have been shown to act as tumor suppressor genes, while others have been shown to be pro-oncogenic. Examples of both of these opposed functions have been identified in Ewing sarcoma.

The FOXO subgroup (consisting of FOXO1, FOXO3A, FOXO4, and FOXO6) are key negative regulators of cell proliferation and survival. They induce cell cycle arrest at G1 (61) and apoptosis and DNA repair (62). They are thus considered bona fide tumor suppressors. For example, in prostate cancer, *FOXO1* is found transcriptionally downregulated and the induction of its expression in prostate cancer cells inhibits cell proliferation and survival (63). In addition, FOXO1 has been also shown to regulate other hallmarks of cancer such as angiogenesis. Thus, FOXO1 loss of function increases blood vessel formation and promotes endothelial cell proliferation and migration (64, 65).

FOXOs transcriptional activity is regulated by changes in their cellular localization, which is mediated by protein kinases such as the serum/glucocorticoid kinase (SGK) and the protein kinase B (AKT) [reviewed in Ref. (66)]. These transcription factors can also undergo different post-translational modifications that regulate their activity including deacetylation mediated by Sirt1 and ubiquitination mediated by Skp2 and Mdm2 (67).

EWS/FLI1 binds to the FOXO1 promoter and represses its expression in Ewing sarcoma cells (68). In accordance with this, FOXO1 is expressed at lower levels in primary Ewing sarcoma as compared to other tissues (16). Induction of FOXO1 in two Ewing sarcoma cells (A673 and SKNMC) resulted in impaired cell proliferation and reduced soft agar colony formation capability, confirming that FOXO1 is a tumor suppressor in Ewing sarcoma and that its inhibition is important for Ewing sarcoma growth. Interestingly, EWS/FLI1 also indirectly regulates the subcellular localization of FOXO1 and thus controls its transcriptional activity. CDK2- (which is upregulated by EWS/FLI1 and acts as a negative regulator of FOXO1 transcriptional activity) and AKT-mediated phosphorylation of FOXO1 cooperate to block its transport to the nucleus thus inhibiting its transcriptional activity. These findings demonstrate that EWS/FLI1 blocks FOXO1 activity at several different levels in Ewing sarcoma cells.

**Figure 3:**
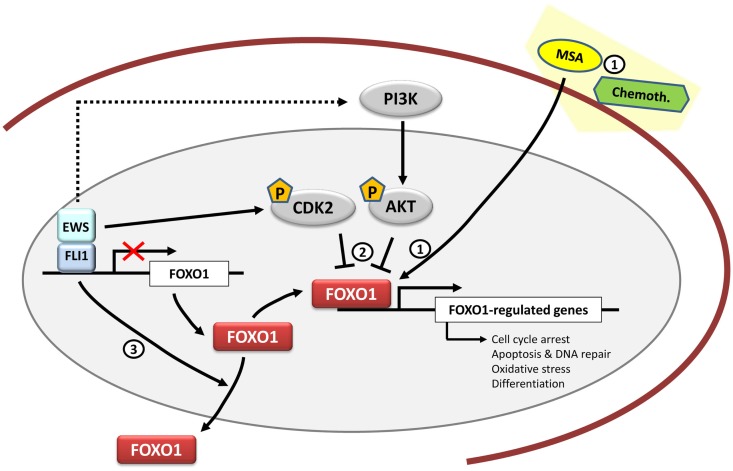
**FOXO1 and therapeutic opportunities in Ewing sarcoma**. *FOXO1* is a direct target gene of EWS/FLI1 and its expression is repressed by EWS/FLI1 in Ewing sarcoma cells. Functional studies have shown that *FOXO1* acts as a tumor suppressor in the Ewing sarcoma cell context. Therapeutically, Methane Sulfonic Acid (MSA) may be used as a potential treatment in synergy with other chemotherapeutic agents such as doxorubicin or etoposide. However, its mechanism of action in Ewing sarcoma is still unknown (1). Also there is still a need to clarify the FOXO1 activities mediated by kinases such as CDK2 and AKT (2) together with the regulation of its subcellular localization (3), and to determine if they may be mediated by EWS/FLI1.

Since FOXO1 acts as a tumor suppressor in Ewing sarcoma, a valuable therapeutic approach can consist in the reactivation of FOXO1 activity. In this regard, methylseleninic acid (MSA), a chemical compound previously shown to reactivate FOXO1 in prostate cancer, was tested in Ewing sarcoma cells (69). Treatment of Ewing sarcoma cells with MSA induced FOXO1 expression in a concentration-dependent manner, which correlated with apoptotic-mediated cell death. This effect was mediated at least in part by FOXO1, since the knockdown of endogenously induced FOXO1 significantly reduced the apoptotic effect of MSA. Notably, administration of MSA in an orthotopic mouse xenotransplantation model significantly reduced tumor growth, suggesting that MSA could be a potential therapeutic approach in Ewing sarcoma. However, it should be taken into account that high concentrations of selenium are usually associated with intoxication, which can make this approach problematic. This means that any potential application of MSA should use effective, low doses, which in combination with conventional chemotherapeutic drugs can reach the desired anti-tumoral effects. Particularly, MSA has already been proved to synergize well with some chemotherapeutic drugs that are frequently used in Ewing sarcoma, such as etoposide or doxorubicin (70) (Figure 3). Since reactivation of FOXO1 in Ewing sarcoma cells has shown to be effective both *in vitro* and *in vivo*, more studies are necessary to understand the mechanism involved in the regulation of FOXO1 expression and its transcriptional activity in order to identify new therapeutic targets.

FOXM1 is another member of the FOX family of transcription factors that contrary to FOXO displays a pro-oncogenic role in cancer. In fact, FOXM1 is one of the most commonly overexpressed genes in solid tumors (71). Initially, FOXM1 was described as a proliferation-specific mammalian transcription factor, expressed in proliferating cells but not in quiescent or terminally differentiated cells. In addition to this, and over the years, FOXM1 has also been implicated in cell migration, invasion, angiogenesis, metastasis, or oxidative stress (72).

Christensen et al. showed that EWS/FLI1 upregulated the levels of FOXM1 in four Ewing sarcoma cell lines, although the mechanism appeared to be indirect (17). In agreement with this, FOXM1 is expressed at high levels in Ewing sarcoma cell lines and primary tumors. In order to characterize the relevance of FOXM1 in Ewing sarcoma pathogenesis, the authors performed FOXM1 knockdown experiments demonstrating that FOXM1 downregulation correlates with a significant reduction in anchorage independent growth.

Interestingly, pharmacological approaches addressed to reduce FOXM1 levels have also been tested in Ewing sarcoma cells with notable results. Thiostrepton, a thiazole antibiotic, has been shown to act as a proteosomal inhibitor (73) and also to physically interact with FOXM1 consequently inhibiting FOXM1 binding to target promoters (74). FOXM1 expression was inhibited by treatment with thiostrepton, which paralleled with an increase in apoptosis in a variety of Ewing sarcoma cell lines (17). Thiostrepton was also shown to inhibit tumor growth in mouse xenograft models (75). Strikingly, in this work, thiostrepton was able to concomitantly inhibit the expression of EWS/FLI1 both at mRNA and protein levels in three Ewing cell lines and in tumors derived from thiostrepton-treated mouse xenograft models (75). Although the mechanism by which thiostrepton promotes EWS/FLI1 downregulation was not characterized, these results suggest that this drug may show greater efficacy in Ewing sarcoma tumors in comparison to other tumors.

**Figure 4:**
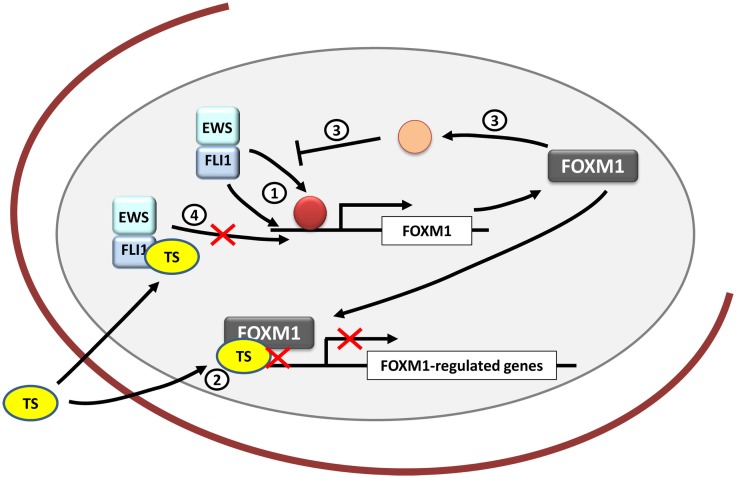
**FOXM1 and therapeutic opportunities in Ewing sarcoma**. *FOXM1* is upregulated by EWS/FLI1 in Ewing sarcoma cells, although it is unknown whether its regulation is direct or indirect (1). Thiostrepton (TS) blocks FOXM1 in Ewing sarcoma cells decreasing their neoplastic features (2). However, the exact mechanism underlying these effects remains unexplained. In addition, FOXM1 has been shown to be capable of inhibiting EWS/FLI1 probably by an indirect mechanism that still needs to be clarified (3). Also TS has been proved to inhibit EWS/FLI1 expression in Ewing cells, although the exact mechanism is still unknown (4).

As stated above, FOXM1 is frequently overexpressed in cancer and takes part in each hallmark of cancer. Consequently it has been argued that targeting FOXM1 could provide an opportunity to treat cancer. It has also been proposed that FOXM1 could be the “Achilles heel” of cancer (76). Taken together, these findings suggest that targeting FOXM1 may be also an opportunity for Ewing sarcoma treatment (Figure 4).

## Secreted Proteins

### Cholecystokinin

Cholecystokinin (CCK) is a neuropeptide that displays a diversity of functions in the organism. It was originally discovered in the gastrointestinal tract, where it mainly regulates pancreatic secretion of digestive enzymes. In addition, CCK is one of the most abundant and widely distributed neuropeptides in the brain, where it modulates intrinsic neuronal excitability and synaptic transmission. CCK is secreted as a prohormone (proCCK) that subsequently undergoes post-translational processing (tyrosine sulfatation, endoproteolytic cleavage, basic residue removal, and C-terminal amidation), resulting in the production of CCK biologically active forms, mainly CCK8 (77).

More than two decades ago, CCK was found to be specifically expressed in a group of human cancer cell lines that included Ewing sarcoma, neuroepithelioma and leiomyosarcomas, as opposed to other tumor cell lines derived from osteogenic sarcomas, rhabdomyosarcoma, melanoma, and SCLC (78). Subsequent studies carried out in tumor specimens confirmed that CCK expression was high in the majority of Ewing sarcomas, whereas in other tumors, CCK-positive cases ranged from 50% in leiomyosarcomas to 0% in medulloblastomas, central primitive neuroectodermal tumors (PNET), neuroblastomas, and rhabdomyosarcomas (79). In agreement with this, a later study demonstrated the presence of proCCK in the supernatant of Ewing sarcoma cell lines in culture, indicating that CCK is actively secreted by Ewing sarcoma cells (80). Interestingly, these authors found high concentrations of proCCK in the plasma of Ewing sarcoma patients but not in patients with other pediatric tumors such as osteosarcoma, neuroblastoma, nephroblastoma, rhabdomyosarcoma or synovial sarcoma. Interestingly, the levels of proCCK in plasma correlated with tumor size and recurrence. In addition, proCCK levels in plasma decreased after chemotherapeutic treatment, concurrently with a reduction in tumor size and in one patient, proCCK levels increased again correlating with tumor recurrence. All together, these results consistently demonstrate that CCK is expressed and secreted at high levels in Ewing sarcoma.

The first data demonstrating a relationship between *CCK* expression and EWS/FLI1 came from studies performed in heterologous systems: ectopic expression of EWS/FLI1 in the RD rhabdomyosarcoma cell line and in HeLa cells (81) upregulated *CCK* mRNA levels. This relationship between EWS/FLI1 and CCK was confirmed in Ewing sarcoma cells. Thus, EWS/FLI1 knockdown in A673 and SK-PN-DW Ewing sarcoma cell lines downregulated CCK mRNA levels, demonstrating that *CCK* expression is dependent on EWS/FLI1. Whether *CCK* is a direct or indirect target of EWS/FLI1 is a question that yet remains to be determined (8). Regarding the functional relevance of CCK in Ewing sarcoma, it was shown that downregulation of *CCK* using a shRNA inducible system, inhibited cell proliferation *in vitro* and tumor growth *in vivo*. In addition, CCK-rich culture media or exogenous CCK-8 was able to stimulate Ewing sarcoma cell proliferation *in vitro*, suggesting that CCK is an autocrine growth factor in Ewing sarcoma cells (8, 82). Unfortunately, to date no studies have been carried out to decipher the mechanisms that underlie this effect in Ewing sarcoma.

The fact that *CCK* is highly expressed in Ewing sarcoma and the observation that it can act as an autocrine growth factor *in vivo* suggest that blocking this autocrine loop, for example, using CCK receptor antagonists, could be of therapeutic interest. CCK and gastrin (a closely related hormone) share two G-protein coupled receptors, named CCKAR and CCKBR that trigger numerous pathways that transmit the mitogenic signal to the nucleus to promote cell proliferation. Whereas CCKA receptors are specific for CCK, CCKB receptors can bind CCK and gastrin with high affinity. Expression of CCK receptors in Ewing sarcoma has been scantly studied with contradictory results. Schaer and Reubi reported a lack of CCK receptors expression in a collection of 11 Ewing sarcoma tumors using autoradiography and ^32^P-labeled CCK-8 as a probe (79). However, more recently it was demonstrated the existence of both CCKA and B receptors mRNA in two Ewing sarcoma cell lines (A673 and SK-PN-DW) and a cohort of ten primary tumors (8).

Treatment of Ewing sarcoma cell lines with devazepide, a specific CCKAR antagonist derived from the benzodiazepine family, induced apoptosis *in vitro* and significantly reduced the tumor growth in a mouse xenograft model (83). However, these effects were observed with IC_50_ values 10,000-fold higher that those necessary to efficiently block the binding of CCK to its CCKA receptor. In addition, one specific antagonist of the CCKB receptor (L365 260) had no effect on Ewing sarcoma cell proliferation or viability (83). These results suggest that in Ewing sarcoma cells there could be an alternative mechanism of action that could involve CCK receptors other than the standard ones, and open the possibility that cell proliferation induced by CCK in Ewing sarcoma cell lines could also be mediated through a yet unknown mechanism.

Regardless of the possibility to block CCK-induced proliferation with specific antagonists, the expression of CCK receptors in tumors can itself be therapeutically useful. In this sense, a model of metastatic medullary thyroid cancer has been successfully used to evaluate the diagnostic and therapeutic potential of radiolabeled gastrin directed to target CCKB receptor-expressing tumors *in vivo* (84). Using this approach, a collection of radiolabeled peptides derived from gastrin and cholecystokinin families showed anti-tumoral activity in xenograft models of medullary thyroid cancer (85) [also extensively reviewed in Ref. (86)]. This means that radiolabeled CCK or other compounds with high affinity for CCK receptors could be useful for diagnosis (i.e., imaging) and perhaps also for the treatment of Ewing sarcoma.

**Figure 5:**
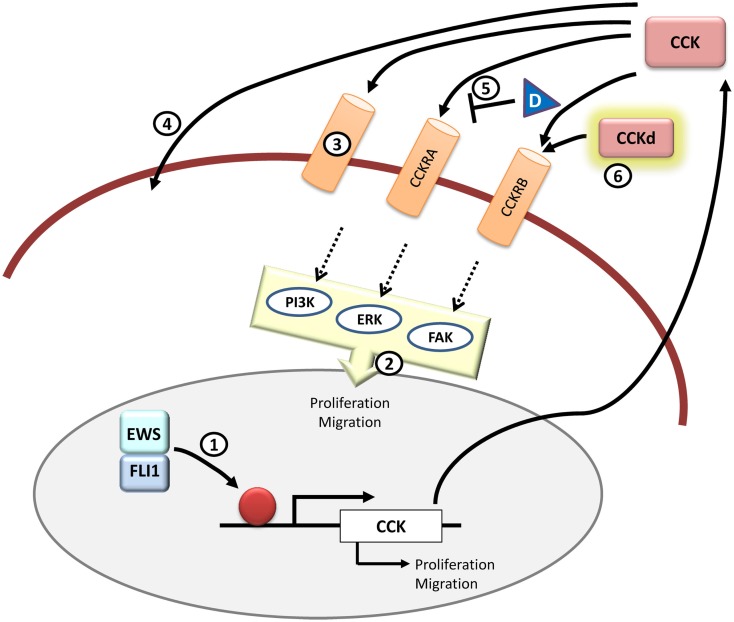
**CCK and therapeutic opportunities in Ewing sarcoma**. *CCK* is an EWS/FLI1 target gene and its expression is elevated in Ewing sarcoma cells. Functionally, inhibition of CCK expression impairs growth and migration in Ewing sarcoma cells. It still remains to be addressed if *CCK* is a direct EWS/FLI1 target or not (1) and which is the exact signaling pathway that takes place once CCK binds to its receptors in the cells (2). Also it is still unknown whether CCK binds exclusively to its canonical CCKRs or if there are other receptors (3) or even if it can enter directly into the cell through some yet unknown mechanism (4). Therapeutically, it may be interesting to further test receptor antagonists other than devazepide (D) that could interfere with CCK binding to its receptors in Ewing sarcoma cells (5). Also, from a diagnosis point of view, it could be useful to test radiolabeled CCK derivatives (CCKd) to be used in imaging technologies (6). All in all, more studies are needed to define the principal components and pathways that are involved in the CCK-autocrine loop.

In summary, although high levels of CCK in Ewing sarcoma tumors were described more than two decades ago, research in this field has been scattered during the last years, and many questions remain unresolved. For example, it is not clear enough what type of CCK receptors are expressed in Ewing sarcoma tumors or the mechanism and intracellular signaling pathways involved in CCK-mediated cell proliferation. Any progress in this regard would help to develop molecules capable of interfering with this autocrine loop (Figure 5).

### LOX

Lysyl oxidase (LOX) (protein lysine-6-oxidase; EC 1.4.3.13) is a member of a family of lysyl oxidases that share the enzyme catalytic domain. This family includes LOX and the LOX-like proteins LOXL1 to 4. These enzymes catalyze lysine-derived covalent crosslinking of collagen and elastin and therefore their function is key for maintaining the structural integrity of the extracellular matrix [extensively reviewed in (87–90)]. LOX is synthesized as a 50-KDa inactive proenzyme (preLOX), which is secreted to the extracellular environment where it is proteolytically processed into a functional 32-KDa LOX enzyme and an 18-KDa propeptide (LOX-PP). Together with the critical role that LOX plays in maintaining the properties of the connective tissues, it has been also shown to play important roles in cancer.

The first evidence of a relationship between LOX and cancer comes from experiments designed to identify genes involved in Ras-mediated transformation of NIH-3T3 mouse fibroblasts (91). Several functional experiments demonstrated that *LOX* had properties that are characteristic of a suppressor gene. Thus, *LOX* antisense cDNA was able to retransform *H-ras*-transformed revertants (92) and confer tumorigenic features to normal rat kidney fibroblasts (NRK-49F) (93).

Since LOX is proteolytically processed into a fragment containing the lysyl oxidase enzymatic activity and an N-terminal propeptide (LOX-PP), experiments were conducted to determine in which of these fragments resided the tumor suppressor activity. Thus, Palamakumbura et al. described for the first time that recombinant LOX-PP was able to inhibit neoplastic transformation features in Ras-transformed mouse fibroblasts such as growth in anchorage independent conditions and Ras-dependent induction of *NF*κ*B* (94). Currently, numerous studies support that the tumor suppressor activity of LOX resides in the 18-kDa propeptide fragment LOX-PP and not in the lysyl oxidase enzyme.

In agreement with its tumor suppressor activity, LOX expression has been reported to be downregulated in many different types of human cancer, such as fibrosarcoma, rhabdomyosarcoma, and melanoma cells (95), lung (96), pancreatic cancer (97), prostate (98), and colorectal cancers (99), which means that LOX expression levels negatively correlate with malignant transformations. By contrast, LOX expression has been also reported to be increased in a number of human cancers [i.e., breast and colon carcinomas (100, 101)] particularly in the metastatic and more aggressive forms of the disease. Interestingly, in these cases, metastatic and invasive properties have been related to the lysyl oxidase activity of LOX, rather than to LOX-PP (100, 101).

The anti-tumor activity of LOX-PP has been demonstrated in various types of tumor cells although the mechanism underlying the tumor suppressor activity of LOX-PP still needs to be clarified. Data obtained until now indicate that LOX-PP can act at different levels, and that the pathways and functions affected can depend of the cancer or cell model studied. For example, in Her-2/neu-transformed NF639 breast cancer cells, ectopic expression of LOX-PP interferes with fibronectin-stimulated tyrosine phosphorylation of cellular proteins involved in integrin signaling, inactivating the focal adhesion kinase (FAK), and consequently diminishes the migratory response (102). In other breast cancer cells driven by Her-2/neu (ERBB2), LOX-PP expression suppressed AKT, ERK, and NFκB activation, as well as cell migration, growth in soft agar and tumor formation in nude mice (103). Moreover, in cells derived from prostate cancer (DU145 and PC-3), LOX-PP blocks FGF-2 binding to the cell, inhibiting MAPK/ERK and PI3K/Akt pathways and blocking serum-stimulated DNA synthesis and cell proliferation (104). On the other hand, LOX-PP decreased the levels of NF-κB and cyclin D1 in Her-2/neu-transformed NF639 breast cancer cells and MIA PaCa-2 pancreatic cancer cells, together with a reduction in migration and growth in soft agar (105). Finally, in PANC-1 pancreatic cancer cells, LOX-PP also impaired AKT and ERK activity and growth in soft agar and cell migration (97).

Recently, a connection between LOX and Ewing sarcoma pathogenesis has been also demonstrated. Thus, EWS/FLI1 knockdown in Ewing sarcoma cells induces the expression of *LOX* indicating that this gene is strongly repressed by EWS/FLI1 in these cells (15). An independent study showed that *LOX* is a direct target of EWS/FLI1 by using ChIP assays (106). In agreement with this, LOX expression was found to be low or undetectable in a group of Ewing sarcoma cell lines and primary tumors (15). Since these data suggested that LOX could act as a tumor suppressor in Ewing sarcoma, functional studies were carried out. Thus, ectopic expression of LOX-PP in the A673 Ewing sarcoma cell line reduced cell proliferation, cell migration, anchorage independent growth, and impaired tumor growth *in vivo*, indicating that it had tumor suppressor activities in this cell, in line with what was observed in other tumors. By contrast, the mature LOX enzyme displayed the opposite effects. Interestingly, when full-length LOX, including LOX enzyme and LOX-PP activities was expressed in A673 cells, the anti-tumor effects prevailed (15). Altogether, these studies indicate that LOX plays an important role in Ewing pathogenesis by acting as a tumor suppressor gene.

The mechanisms involved in LOX-PP-mediated suppression in Ewing sarcoma have only been partially studied. In one study, ectopic expression of LOX-PP showed to impair ERK signaling pathway, whereas the PI3K/AKT pathway remained unaffected (15). Interestingly, in this work, an analysis of the gene expression profile induced by LOX-PP expression in the A673 Ewing cell line showed that a significant proportion of the genes affected belonged to pathways involved in cell proliferation and cell cycle control. Given the impact that LOX-PP expression has on tumorigenesis, it is necessary to extend these studies in order to characterize in more detail the pathways that may be affected by the exposition of Ewing sarcoma cells to LOX-PP, and particularly to determine which specific growth factor pathways could be affected by LOX-PP.

Other interesting aspect that remains to be determined is the identification of the proteins that interact with LOX-PP in Ewing sarcoma cells. In other cell types, LOX-PP has been shown to interact with a number of proteins such as Hsp70, c-Raf or CIN85 (107), so it would be interesting to identify and characterize LOX-PP partners in the specific Ewing sarcoma cell context and to elucidate their role in LOX-PP mediated tumor suppression.

The fact that LOX-PP acts as a tumor suppressor gene in cancer, and specifically in Ewing sarcoma, invites to assess the therapeutic value of LOX-PP. The easiest strategy is to evaluate the effect of the administration of LOX-PP on tumor cells. Thus, recombinant LOX-PP has been used to ascertain its therapeutic potential in several cancer types both *in vitro* and *in vivo* (94, 97, 102–105, 108, 109). In all cases, exogenous LOX-PP reduced tumor cells growth, supporting the therapeutic usefulness of this strategy. Interestingly, in one study, the combination of LOX-PP with the chemotherapeutic agent doxorubicin in breast and pancreatic cancer cells *in vitro* showed an enhanced cytotoxic effect of doxorubicin when the cells were first sensitize by incubation with LOX-PP (105). These results mean that even if LOX-PP is not capable of inducing complete cell death by itself, it could potentially sensitize cancer cells to standard therapies thus allowing to lower the doses and adverse side effects associated to conventional chemotherapy and radiotherapy. At the moment, there are no data about the effect of exogenous administration of LOX-PP, alone or in combination with chemotherapeutic drugs, on Ewing sarcoma cells. These preclinical studies are therefore needed to test if this strategy can represent a promising line of research in order to find new therapeutic approaches to treat Ewing sarcoma patients.

Since *LOX* expression, and thus LOX-PP, is downregulated in Ewing sarcoma cells by EWS/FLI1 (15, 106), other therapeutic approach could be the induction of LOX expression, and thus LOX-PP, in these cells. In this line, it has been proposed that EWS/FLI1 binds to *LOX* promoter and downregulates *LOX* expression by recruiting the NuRD transcriptional repressor complex containing the HDACs and LSD1 associated proteins. Interestingly, the use of HDACs inhibitors (vorinostat/SAHA) or LSD1 inhibitors (HCI-2509) induced an increase in the levels of LOX mRNA in A673 Ewing sarcoma cells, which suggest that the anti-tumor effect of these drugs could be mediated, at least in part, by the upregulation of *LOX* (106). However, induction of *LOX* expression to achieve an increased production of anti-tumorigenic LOX-PP in Ewing sarcoma cells may not be as beneficial as expected: while induction of *LOX* expression would cause an increase in LOX-PP, it also would produce an increase in the production of the LOX mature enzyme, which has been showed to be pro-oncogenic in Ewing sarcoma cells and other tumors (15, 100, 101).

Other opportunities for therapeutic interventions could be derived from the identification and characterization of LOX-PP interactions with other proteins, mainly intracellular proteins involved in cell signaling and regulation of tumorigenic processes. Biochemical studies have shown that LOX-PP is an intrinsically disordered protein (110). These proteins are expected to participate in signaling processes due to their capability to adopt interconverting structures and to interact with their partners, and have been proposed to be potential drug targets (111). Thus, characterization of the exact motifs that are involved in LOX-PP interactions can open the door to the identification of targetable proteins and the design of small molecules capable to reproduce the effect of LOX-PP.

**Figure 6:**
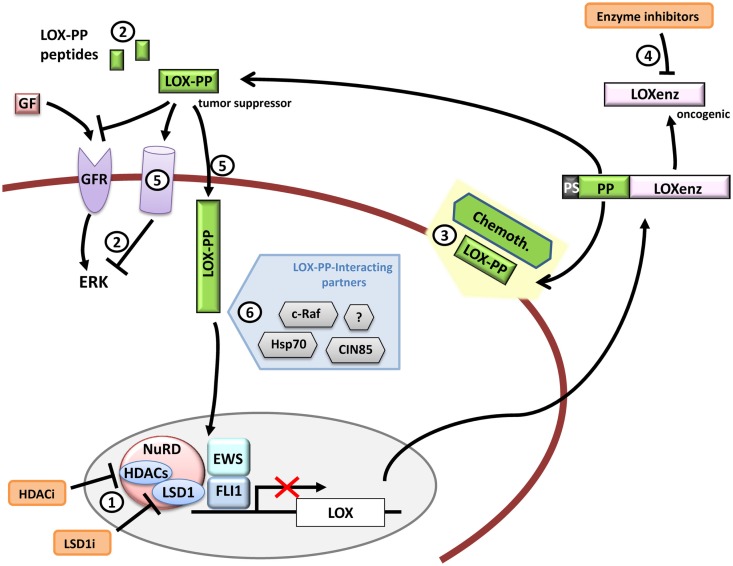
**LOX and therapeutic opportunities in Ewing sarcoma**. *LOX* expression is repressed by EWS/FLI1 in Ewing sarcoma cells. Functional studies have demonstrated that LOX acts as a tumor suppressor gene in Ewing sarcoma and that its activity resides in its propeptide domain (LOX-PP). Therapeutic opportunities could include for example (1) *LOX* de-repression by targeting repression complexes that interact with EWS/FLI1 at the *LOX* promoter, (2) administration of LOX-PP or LOX-PP active derived peptides to block ERK signaling alone or in combination with traditional chemotherapy (3) or blocking the LOXenz fraction activity (4). The mechanisms through which LOX-PP exerts its anti-tumor activity are largely unknown, especially in Ewing sarcoma. For instance, it is currently unknown if LOX-PP specific receptors (5) (intracellular or transmembrane) are necessary to produce its anti-tumor activities or if the different LOX-PP-interacting proteins may interfere with its activity in Ewing sarcoma (6). Any advance in these aspects could provide new clues to design new therapeutic tools.

In summary, LOX, and more specifically LOX-PP, has been showed to have anti-tumorigenic properties, which could be exploited to treat cancer cells. Regarding Ewing sarcoma, it is yet more than necessary to characterize the pathways involved in LOX-PP mediated tumor-suppression, in particular the identification of the protein interactions that mediate this response, in order to identify key factors that could provide new therapeutic targets (Figure 6).

## Conclusion

Ewing sarcoma is driven by EWS/FLI1, which is a protein generated by a tumor-specific aberrant translocation. Although it may seem like a perfect target for therapeutic applications, directed therapies toward it have failed to reach the clinic (112). For this reason, the identification of EWS–FLI target genes and their role in tumor signaling networks have been addressed in the last years, and some excellent reviews have assessed this topic (4, 113, 114).

This review is focused on the EWS/FLI1 downstream regulatory network, particularly on EWS/FLI1 up- and down-regulated target genes on which the study of potential targeted therapies could be of clinical interest. Also, we stated some current questions regarding pathways and unknown mechanisms underlying the functional effects of these genes in Ewing sarcoma that still remain unresolved and could help find key clues for the future studies of this disease. There are plenty of mechanisms regarding EWS/FLI1 target genes that are still unknown and a deeper knowledge on them could potentially lead to the development of more specific and less toxic therapies in Ewing sarcoma.

## Author Contributions

FC-A and JA wrote the manuscript and designed the figures. JA corrected and supervised the manuscript.

## Conflict of Interest Statement

The authors declare that the research was conducted in the absence of any commercial or financial relationships that could be construed as a potential conflict of interest.
